# Selenium Nanoparticles with Prodigiosin Rescue Hippocampal Damage Associated with Epileptic Seizures Induced by Pentylenetetrazole in Rats

**DOI:** 10.3390/biology11030354

**Published:** 2022-02-23

**Authors:** Naif E. Al Omairi, Ashraf Albrakati, Khalaf F. Alsharif, Abdulraheem S. Almalki, Walaa Alsanie, Zakaria Y. Abd Elmageed, Dalia Zaafar, Maha S. Lokman, Amira A. Bauomy, Saied K. Belal, Mohamed M. Abdel-Daim, Ahmed E. Abdel Moneim, Hussain Alyami, Rami B. Kassab

**Affiliations:** 1 Department of Internal Medicine, College of Medicine, Taif University, P.O. Box 11099, Taif 21944, Saudi Arabia; naifalomairi78@gmail.com (N.E.A.O.); hmyami@tu.edu.sa (H.A.); 2 Department of Human Anatomy, College of Medicine, Taif University, P.O. Box 11099, Taif 21944, Saudi Arabia; sbelal@tu.edu.sa; 3 Department of Clinical Laboratory Sciences, College of Applied Medical Sciences, Taif University, P.O. Box 11099, Taif 21944, Saudi Arabia; alsharif@tu.edu.sa (K.F.A.); w.alsanie@tu.edu.sa (W.A.); 4 Department of Chemistry, Faculty of Science, Taif University, Taif 21974, Saudi Arabia; almalki.a@tu.edu.sa; 5 Department of Pharmacology, Edward Via College of Osteopathic Medicine, University of Louisiana at Monroe, Monroe, LA 71203, USA; zelmageed@ulm.vcom.edu; 6 Department of Pharmacology and Toxicology, Faculty of Pharmacy, Modern University for Technology and Information, Cairo 11311, Egypt; dalia.zaffar@pharm.mti.edu.eg; 7 Biology Department, College of Science and Humanities, Prince Sattam bin Abdul Aziz University, Alkharj 11942, Saudi Arabia; ms.hussein@psau.edu.sa; 8 Department of Zoology and Entomology, Faculty of Science, Helwan University, Cairo 11795, Egypt; aest1977@hotmail.com (A.E.A.M.); ramikassab@mail.muni.cz (R.B.K.); 9 Department of Science Laboratories, College of Science and Arts, Qassim University, ArRass 52719, Saudi Arabia; amiraanwar1@gmail.com; 10 Department of Pharmaceutical Sciences, Pharmacy Program, Batterjee Medical College, P.O. Box 6231, Jeddah 21442, Saudi Arabia; abdeldaim.m@vet.suez.edu.eg; 11 Pharmacology Department, Faculty of Veterinary Medicine, Suez Canal University, Ismailia 41522, Egypt; 12 Biology Department, Faculty of Science and Arts, Al-Baha University, Al-Mukhwah 65554, Saudi Arabia

**Keywords:** prodigiosin, selenium nanoparticles, epilepsy, oxidative stress, neuroinflammation, apoptosis, neurotransmission

## Abstract

**Simple Summary:**

Epilepsy is a chronic neurological disease characterized by neuronal hyper electrical activity and the development of unprovoked seizures. Although several antiepileptic drugs are currently available, their application is associated with undesirable adverse effects. In an attempt to find a novel antiepileptic medication with minimum side effects, we have investigated the potential neuroprotective activity of prodigiosin, a red pigment produced by bacterial species that have important pharmaceutical and biological activities biosynthesized with selenium formulation (SeNPs-PDG) against a murine epileptic model mediated by pentylenetetrazole. The main recorded findings revealed that SeNPs-PDG delayed the onset of epileptic seizures and decreased their duration significantly. Additionally, SeNPs-PDG prevented hippocampal cell loss, oxidative stress, neuroinflammation, restored the balance between excitatory and inhibitory neurotransmitters, and notably normalized the monoaminergic and cholinergic transmission. These promising findings indicate that SeNPs-PDG might serve as a naturally derived anticonvulsant agent due to their active antioxidant, anti-inflammatory, anti-apoptotic, and neuromodulatory properties.

**Abstract:**

Background: Prodigiosin (PDG) is a red pigment synthesized by bacterial species with important pharmaceutical and biological activities. Here, we investigated the neuroprotective and anticonvulsant activities of green biosynthesized selenium formulations with PDG (SeNPs-PDG) versus pentylenetetrazole (PTZ)-induced epileptic seizures. Methods: Rats were assigned into six experimental groups: control; PTZ (60 mg/kg, epileptic model); sodium valproate (200 mg/kg) + PTZ; PDG (300 mg/kg) + PTZ; sodium selenite (0.5 mg/kg) + PTZ; and SeNPs-PDG (0.5 mg/kg) + PTZ. The treatment duration is extended to 28 days. Results: SeNPs-PDG pre-treatment delayed seizures onset and reduced duration upon PTZ injection. Additionally, SeNPs-PDG enhanced the antioxidant capacity of hippocampal tissue by activating the expression of nuclear factor erythroid 2–related factor 2 and innate antioxidants (glutathione and glutathione derivatives, in addition to superoxide dismutase and catalase) and decreasing the levels of pro-oxidants (lipoperoxidation products and nitric oxide). SeNPs-PDG administration inhibited inflammatory reactions associated with epileptic seizure development by suppressing the production and activity of glial fibrillary acidic protein and pro-inflammatory mediators, including interleukin-1 beta, tumor necrosis factor-alpha, cyclooxygenase-2, inducible nitric oxide synthase, and nuclear factor kappa B. Moreover, SeNPs-PDG protected against hippocampal cell loss following PTZ injection by decreasing the levels of cytosolic cytochrome c, Bax, and caspase-3 and enhancing the expression of anti-apoptotic Bcl-2. Interestingly, SeNPs-PDG restored the PTZ-induced imbalance between excitatory and inhibitory amino acids and improved monoaminergic and cholinergic transmission. Conclusions: These promising antioxidative, anti-inflammatory, anti-apoptotic, and neuromodulatory activities indicate that SeNPs-PDG might serve as a naturally derived anticonvulsant agent.

## 1. Introduction

Epilepsy is a neurodegenerative condition characterized by neuronal hyperexcitability accompanied by repetitive and unprovoked convulsions [1]. Epileptic seizures are primarily accompanied by cerebrovascular dysfunction, brain injury, tumors, and hypoxia [2]; however, the underlying mechanisms that develop epileptic seizures remain unclear. An imbalance between neuronal excitation and inhibition, oxidative insults, inflammation, neuronal cell loss, and dysregulated neurotransmission are hypothesized to play fundamental roles in the development of epilepsy [3,4]. Oxidative stress and the resulting overproduction of reactive oxygen (ROS) and nitrogen species (RNS), combined with the depletion of the cellular antioxidant capacity, are important hallmarks of epileptogenesis [5]. Nuclear factor erythroid 2–related factor 2 (*Nrf2*) is a transcriptional factor that modulates the expression of several proteins involved in cellular antioxidant protection and detoxification processes, such as heme oxygenase-1 (HO-1), superoxide dismutase (SOD), and glutathione (GSH)-derived enzymes [6]. *Nrf2* activation in response to ROS overproduction is associated with the initiation and progression of epileptic seizures, and *Nrf2* has been considered a prospective target for antiepileptic drugs [6]. Oxidative stress-mediated neuronal cell death plays a crucial role in epilepsy pathogenesis [7]. Accumulating evidence suggests that seizure propagation is associated with a neuroinflammatory state characterized by the activation of neuroglial cells that produce excessive quantities of pro-inflammatory mediators, including glial fibrillary acidic protein (GFAP), interleukin-1 beta (IL-1β), tumor necrosis factor-alpha (TNF-α), cyclooxygenase-2 (Cox-2), inducible nitric oxide synthase (iNOS), and nuclear factor kappa B (NF-κB), resulting in neuronal hyperexcitability and cell death [8]. Disruptions in brain monoamines, free amino acids, and acetylcholine transmission have also been reported during epileptogenesis [9].

Currently available antiepileptic medications cause many adverse events, including memory disturbances, gastrointestinal distress, osteoporosis, depression, fatigue, nausea, and dizziness [10]. Formulating efficient anticonvulsant drugs with minimal side effects remains an urgent and unmet need. Progress in nanotechnology has led to the development of various metal-based nanoformulations that have recently been used to treat neurodegenerative disorders [11]. The effects of nanoparticles (NPs) on targeted tissues depend on the NP size, shape, concentration, and type, in addition to the tissue structure and duration of exposure [12]. Selenium nanoparticles (SeNPs) are absorbed more effectively than Se (IV), and their relatively small sizes allow them to cross membrane barriers and accumulate in tissues, leading to increased reactivity [13]. SeNPs have recently been used to prevent and treat neurological conditions due to their relatively high bioavailability, low occurrence of adverse effects, and potential therapeutic activities [14], which include potent antioxidant, anti-inflammatory, anti-apoptotic, and neuromodulatory activities, as reported when administered to an epileptic rat model [4].

Prodigiosin (PDG) is a red dye naturally produced by various bacterial species and is characterized by a typical pyrrolyl pyrromethene skeleton [15]. PDG has several biological and pharmaceutical activities, including antioxidant, antimalarial, antibacterial, anticancer, anti-inflammatory, and immunosuppressive [16]. To the best of our knowledge, the antiepileptic properties of PDG, either alone or when delivered using SeNPs, have not yet been investigated. We aimed to explore the potential antiepileptic effects of biosynthesized SeNPs with PDG (SeNPs-PDG) against pentylenetetrazole (PTZ)-induced acute epileptic seizures by examining the oxidative, inflammatory, and apoptotic profiles and exploring neurochemical and histopathological changes in the hippocampus.

## 2. Materials and Methods

### 2.1. Chemicals

PTZ was purchased from Sigma Chemical Co. (St. Louis, MO, USA). The synthesis of SeNPs-PDG was performed as follows. 10 mL sodium selenite (Na_2_SeO_3_, 10 mM) was swirled with 10 mL PDG (3.5 mg/mL). Under stirring magnetic conditions overnight, the obtained mixture (SeNPs-PDG) was lyophilized using a vacuum freeze dryer (LabconcoFreezone 4.5 Liter Freeze Dry System, Marshall Scientific, Hampton, NH, USA). The developed powder was used in the current investigation. Our recent report showed that the obtained nanoformulation (SeNPs-PDG) are characterized with 109.3 average diameters and −13.9 mV as a mean zeta potential record [17]. In addition, TEM analysis revealed that the size of the developed nanoformulation is smaller than 200 nm (Figure S1).

### 2.2. Experimental Animals and Study Design

Ten-week-old male albino Wistar rats (180–200 g) were sourced from the animal facilities of King Fahd Center for Medical Research, King Abdulaziz University, Jeddah, Saudi Arabia. Under standard lab conditions, rats were placed in wire polypropylene cages (12-h light/dark cycle; 25 ± 2 °C). Standard diet and water were provided ad libitum. The rats were acclimatized for seven days prior to the start of the study. All experimental protocols were approved by the Committee of Research Ethics for Laboratory Animal Care, Taif University (approval no. 43-068).

Rats were randomly distributed into six groups (*n* = 9/group):

Group I, control group: Rats received a daily oral dose of 0.9% NaCl for 28 days. On Day 28, rats were injected intraperitoneal (i.p.) with 0.9% NaCl, 1 h after the oral administration of saline.

Group II, PTZ-treated group (PTZ): Rats received a daily oral dose of normal saline for 28 days. On Day 28, animals received a single i.p. injection of PTZ (60 mg/kg) to induce acute epileptic seizures, 1 h after the oral administration of saline, as described by Abdel-Rahman et al. [18].

Group III, sodium valproate (VPA) + PTZ-treated group (VPA + PTZ): Rats received a daily oral dose of VPA (200 mg/kg) for 28 days, based on the methodology described by Arafa et al. [19]. On Day 28, the rats received a single i.p. injection of PTZ (60 mg/kg), 1hafter VPA administration.

Group IV, PDG+ PTZ-treated group (PDG+PTZ): Rats received a daily oral dose of PDG (300 mg/kg, [20]) for 28 days. On Day 28, animals received a single i.p. injection of PTZ (60 mg/kg), 1 h after the oral administration of PDG.

Group V, Na_2_SeO_3_ + PTZ-treated group (Na_2_SeO_3_ + PTZ): Rats received a daily oral dose of Na_2_SeO_3_ (0.5 mg/kg) for 28 days, as previously described [21]. On Day 28, animals received a single i.p. injection of PTZ (60 mg/kg), 1 h after the oral administration of Na_2_SeO_3_.

Group VI, SeNPs-PDG + PTZ-treated group (SeNPs-PDG + PTZ): Rats received a daily oral dose of SeNPs-PDG (0.5 mg/kg) for 28 days, as described by Yuan et al. [4]. On Day 28, animals received a single i.p. injection of PTZ (60 mg/kg), 1 h after the oral administration of SeNPs-PDG.

PTZ, Na_2_SeO_3_, SeNPs-PDG, and VPA were administered after dissolving in normal saline. Rats were euthanized one day after the last dose. The hippocampus was immediately separated and washed with isotonic saline. Hippocampal tissue was homogenized in ice-cold 10 mM phosphate buffer (pH 7.4) to produce a 10% (*w*/*v*) homogenate for biochemical evaluations. To assess monoamine and amino acid-based neurotransmitters, hippocampal tissue was homogenized in 75% HPLC grade methanol (10% *w*/*v*) and centrifuged for 10 min at 4000 rpm, and the supernatant was subjected to HPLC. To examine histopathological changes, hippocampal tissues were fixed in 10% neutral buffered formalin, and the CA1 region was assessed for histopathological changes. The methodology described by Lowry et al. was used to estimate the protein level in the hippocampal tissue [22].

### 2.3. Behavioral Record

Animals were monitored immediately after PTZ injection for forty minutes, and a seizure index score was determined based on the revised Racine scale [23] as follow:

0 = no behavioral change, 1 = Whisker trembling, 2 = Facial jerking, 3 = Neck jerking, 4 = Clonic seizures, 5 = Lying on side and wild jumping and 6= death. Additionally, the duration of seizures was recorded. 

### 2.4. Estimation of Redox Status

#### 2.4.1. Oxidative Stress Index Evaluation

Malondialdehyde (MDA) was assessed to reflect the level of lipid peroxidation using the thiobarbituric acid method described by Ohkawa et al. [24]. Dye formation following the addition of Griess reagent was measured at 540 nm to assess the nitric oxide (NO) level in the hippocampus, as previously described [25]. GSH levels were estimated using Ellman’s reagent, and the yellow chromogen was estimated at 412 nm, as previously described [26].

#### 2.4.2. Antioxidant Activity Assessment

The hippocampal activity of glutathione reductase (GR) was investigated as described by Factor et al. [27]. Glutathione peroxidase (GPx) was assessed according to the procedures described by Paglia and Valentine [28]. Hippocampal catalase (CAT) activity was estimated according to the method described by Aebi [29]. Hippocampal SOD activity was determined at 480 nm using the technique described by Misra and Fridovich [30]. 

### 2.5. Estimation of Inflammatory Cytokines

For the determination of hippocampal levels of different pro-inflammatory mediators, commercial enzyme-linked immunosorbent assay (ELISA) kits were utilized for Cox-2, IL-1β, TNF-α, and NF-κB (Novus Biologicals, Centennial, CO, USA; catalog numbers: NB600-971, NBP1-92702, NBP1-92681, and NB100-2176, respectively), according to the manufacturer’s instructions.

### 2.6. Assessment of Pro-Apoptotic and Anti-Apoptotic Proteins

Hippocampal apoptotic proteins were assayed using ELISA kits for Bax and Bcl-2 (BioVision, Inc., Milpitas, CA, USA; catalog numbers E4513 and CSB-E08854r, respectively). A colorimetric kit was used to measure caspase-3 activity (Sigma-Aldrich: CASP3C-1KT). Cytochrome c and brain-derived neurotrophic factor (BDNF) were determined using ELISA kits sourced from Abcam (catalog numbers: ab210575 and ab213899, respectively). All assays were performed according to the manufacturer’s instructions.

### 2.7. Assessment of Acetylcholinesterase, Monoamines, and Free Amino Acid Neurotransmitters

The hippocampal activity of acetylcholinesterase (AChE) was assessed according to the procedure described by Elman et al. [31]. AChE activity was determined based on the yellow color developed following the addition of thionitrobenzoic acid, measured at 412 nm. HPLC reports and chromatograms were obtained using the data acquisition program (ChemStation, Dayton, OH, USA). Hippocampal samples were subjected to a solid-phase extraction using a CHROMABOND column (Cat. No. 730031) to remove trace elements and lipids, and the NH2 phase was retrieved. The NH2 phase was injected into an AQUA column (150 mm; 5 µm; C18, Phenomenex, CA, USA). After 12 min, dopamine (DA), norepinephrine (NE), and serotonin (5-HT) were separated. Each monoamine position and concentration in the sample was identified by comparing the resulting chromatogram with that of the corresponding standard (Sigma Chemical Co., St. Louis, MO, USA). According to Pagel et al. [32], the concentration of each monoamine was quantified relative to total brain tissue (µg/g). Glutamate and γ-aminobutyric acid (GABA) levels were estimated using the precolumn phenylisothiocyanate derivatization technique described by Heinrikson and Meredith [33].

### 2.8. Real-Time PCR

The RNeasy Plus Mini-kit extracted total RNA from hippocampal tissue (Qiagen, Valencia, CA, USA). A cDNA synthesis kit was used to synthesize first-stranded cDNA (Bio-Rad, CA, USA), then amplified in three technical replicates using Power SYBR Green (Life Technologies, CA, USA) and measured using an Applied Biosystems 7500 instrument (Roche Molecular Systems, Inc., Foster City, CA, USA). The PCR conditions were 95 °C for 4 min, followed by 40 cycles of 94 °C for 60 s and 55 °C for 60 s, and a final extension at 72 °C for 10 min. After amplification, cycle numbers at the linear amplification threshold (Ct) for the reference gene *Gapdh* were determined for each sample, and relative gene expression was determined using the comparative Ct method [34]. The Primer-Blast program provided by the National Center for Biotechnology Information (NCBI) was used to design PCR primers for *Nrf2*, *Nos2*, *Creb1*, and *Gapdh*, which were then synthesized by Jena Bioscience GmbH (Jena, Germany). Table 1 shows the primer sequences and accession numbers for the genes examined in the current study.

### 2.9. Histopathological and Immunohistochemical Examinations

Hippocampal tissue was fixed in 10% neutral buffered formalin, dehydrated, and paraffinized at room temperature for 24h, followed by sectioning (4–5 µm). Sections were stained with hematoxylin and eosin for light microscopy analysis.

For GFAP immunohistochemistry, brain sections were incubated in 10% H_2_O_2_ for 30 min to eliminate endogenous peroxidase activity and blocked for one hour with 10% normal goat serum at room temperature. Sections were then incubated in primary anti-GFAP antibodies (Abcam, Cambridge, MA, USA; 1/1000) for 24 h at 4 °C. Antibody detection was performed using the Histostain-Plus Bulk kit (Invitrogen, Camarillo, CA, USA) against rabbit IgG, and finally, 3,3′-diaminobenzidine was used for visualization. A Nikon microscope was used to capture photomicrographs at 400× magnification (Eclipse E200-LED, Tokyo, Japan). GFAP expression was estimated by counting GFAP (+) cells in random sections of each rat.

### 2.10. Statistical Analysis

Data are expressed as the mean ± standard deviation (SD). Measurements were examined by one-way analysis of variance (ANOVA), followed by Duncan’s post hoc test, using the statistical package SPSS version 23.0. *p*-values < 0.05 were considered significant.

## 3. Results

### 3.1. Induction of Epileptic Seizures Using PTZ

PTZ injection (60 mg/kg, i.p.) induced tonic, myoclonic, and generalized seizures as assessed using the Racine scale (Table 2). Similar to the standard antiepileptic drug (VPA), the pre-administration of PDG alone or SeNPs-PDG for 28 consecutive days significantly reduced seizure duration, flexion, extension, and clonus, indicating an anticonvulsant activity.

### 3.2. SeNPs-PDG Administration Induces Antioxidant Effects Following PTZ Injection

**Figure 1:**
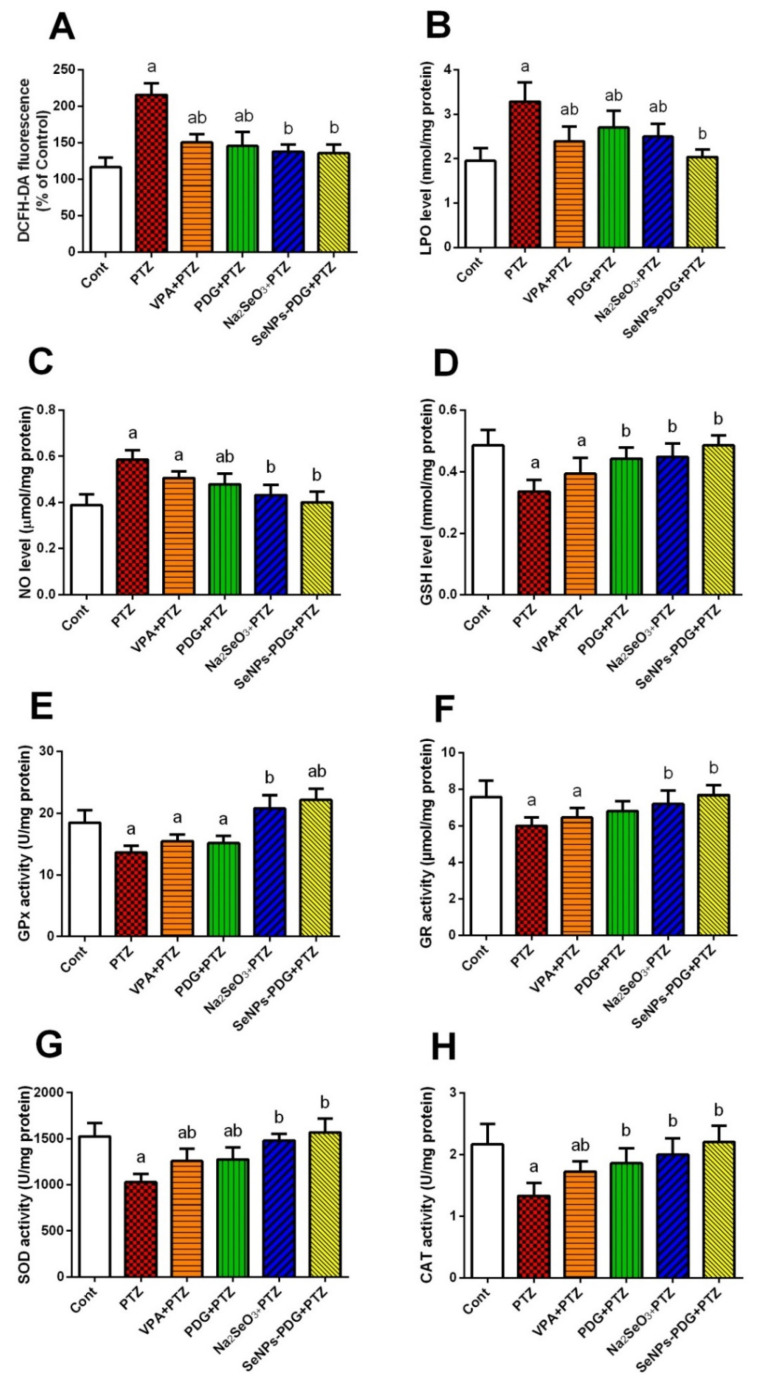
Effects of orally administered prodigiosin (PDG), sodium selenite (Na_2_SeO_3_), and green biosynthesized SeNPs with PDG (SeNPs-PDG) on the hippocampal levels of (**A**) reactive oxygen species (ROS), (**B**) malondialdehyde (MDA), (**C**) nitric oxide (NO), (**D**) glutathione (GSH), (**E**) glutathione peroxidase (GPx), (**F**) glutathione reductase (GR), (**G**) superoxide dismutase (SOD), and (**H**) catalase (CAT) following pentylenetetrazole (PTZ)-induced epileptic seizures. ^a^ and ^b^ denote significant differences (*p* < 0.05) compared with the untreated control and PTZ-injected groups, respectively. All records are presented as the mean ± standard deviation (SD).

The levels of both pro-oxidants and antioxidant molecules were estimated to evaluate the hippocampal redox status in all experimental groups. A single PTZ injection at 60 mg/kg provoked an oxidative challenge in hippocampal slices, characterized by high ROS levels; increased lipid peroxidation, determined by the MDA levels; and the formation of NO. An accompanying decrease in the cellular antioxidant capacity was also observed, including decreased levels of GSH, GPx, GR, SOD, and CAT activities compared with control levels. Expectedly, all applied treatments, especially SeNPs-PDG, were able to restore the balance between pro-oxidants and cellular antioxidants compared with the epileptic group (Figure 1).

**Figure 2:**
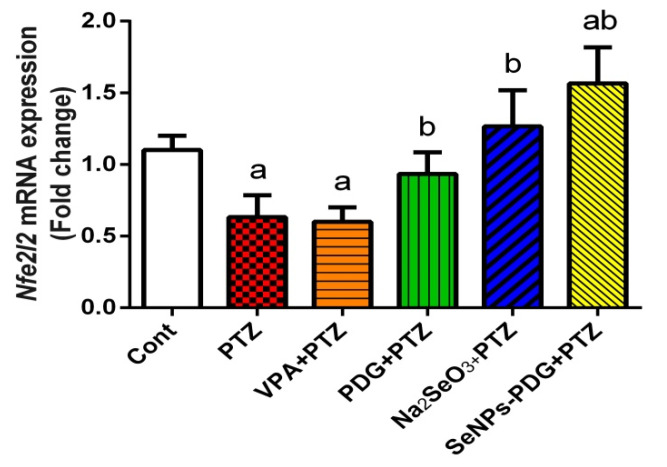
Effects of orally administered prodigiosin (PDG), sodium selenite (Na_2_SeO_3_), and green biosynthesized SeNPs with PDG (SeNPs-PDG) on the mRNA expression levels of *Nrf2* in hippocampal tissue following pentylenetetrazole (PTZ)-induced epileptic seizures. ^a^ and ^b^ denote significant differences (*p* < 0.05) compared with the untreated control and PTZ-injected groups, respectively. All records are presented as the mean ± standard deviation (SD).

To elucidate the antioxidant impacts of SeNPs-PDG against oxidative insults following the PTZ injection, the mRNA expression level of *Nrf2* was estimated in the hippocampal tissue. The results indicated that the PTZ injection downregulated the mRNA expression of *Nrf2* compared with its expression levels in the control group. The pre-administration of SeNPs-PDG upregulated *Nrf2* expression levels above those in the PTZ group, which may mediate the increased cellular antioxidant capacity observed for the administration of SeNPs-PDG against oxidative stress produced by PTZ (Figure 2).

### 3.3. SeNPs-PDG Administration Induces Anti-Inflammatory Effects Following PTZ Injection

**Figure 3:**
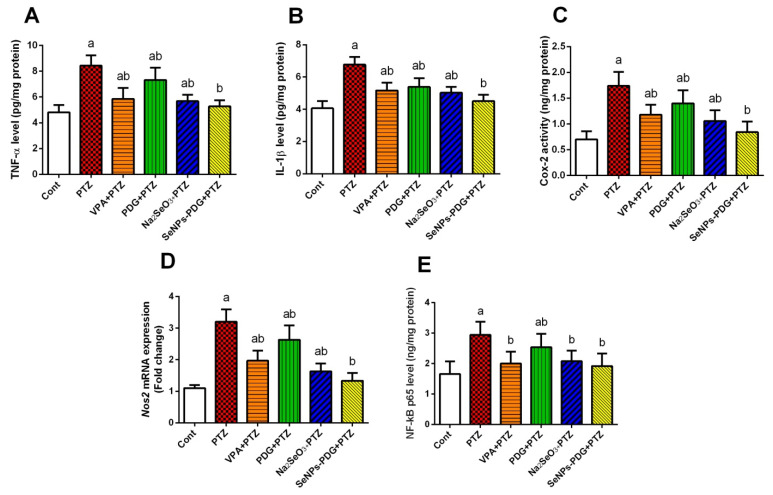
Effects of orally administered prodigiosin (PDG), sodium selenite (Na_2_SeO_3_), and green biosynthesized SeNPs with PDG (SeNPs-PDG) on the levels of inflammatory mediators, including (**A)** TNF-α, (**B**) IL-1β, (**C**) Cox-2, (**D**) *Nos2* mRNA expression, and (**E**) NF-κB in hippocampal tissue following pentylenetetrazole (PTZ)-induced epileptic seizures. ^a^ and ^b^ denote significant differences (*p* < 0.05) compared with the untreated control and PTZ-injected groups, respectively. All results are presented as the mean ± standard deviation (SD).

The levels of TNF-α, IL-1β, Cox-2, and NF-κB and the mRNA expression level of *Nos2* were estimated to investigate the potential anti-inflammatory effects associated with SeNPs-PDG administration in response to PTZ-induced neuroinflammation. The PTZ treatment group demonstrated a significant elevation of all measured pro-inflammatory biomarkers in hippocampal tissue compared with the control group. By contrast, the SeNPs-PDG pretreated group displayed normalized levels of these pro-inflammatory mediators compared with the PTZ-treated group. These results indicated the promising anti-inflammatory effects of SeNPs-PDG against PTZ-mediated neuronal inflammation (Figure 3).

### 3.4. SeNPs-PDG Administration Inhibits Neuronal Loss and Modulates Neuronal Maintenance in Hippocampal Tissue Following PTZ Injection

**Figure 4:**
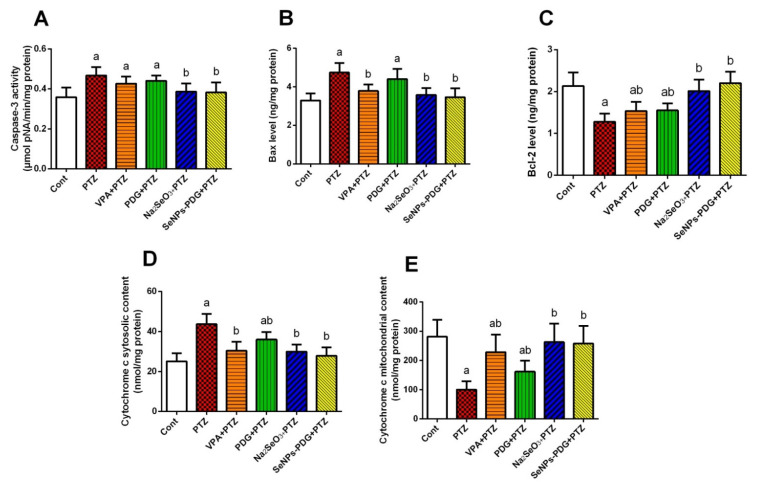
Effects of orally administered prodigiosin (PDG), sodium selenite (Na_2_SeO_3_), and green biosynthesized SeNPs with PDG (SeNPs-PDG) on the levels of apoptosis markers, including (**A**) caspase-3, (**B**) Bax, (**C**) Bcl-2, (**D**) mitochondrial cytochrome c, and (**E**) cytosolic cytochrome c in hippocampal tissue following pentylenetetrazole (PTZ)-induced epileptic seizures. ^a^ and ^b^ denote significant differences (*p* < 0.05) compared with the untreated control and PTZ-injected groups, respectively. All results are presented as the mean ± standard deviation (SD).

Neuronal apoptosis was assessed in the hippocampal tissues of all experimented groups by evaluating the levels of Bax, caspase-3, Bcl-2, and both cytosolic and mitochondrial cytochrome c. The results revealed that PTZ injection enhanced neuronal apoptosis in the examined brain tissue, as indicated by the elevation of pro-apoptotic proteins, including Bax and caspase-3, and the diminished expression levels of the anti-apoptotic protein Bcl-2 relative to control rats. Additionally, the PTZ injection enhanced the release of cytochrome c from the mitochondria into the cytoplasm, associated with increased neuronal apoptosis in the hippocampal tissue and the development of epileptic seizures. Supplementation with both PDG and SeNPs-PDG protected hippocampal tissue by blocking the release of mitochondrial cytochrome c, suppressing neuronal apoptosis, which was accompanied by decreased levels of Bax and caspase-3 and increased levels of Bcl-2 compared with those in the PTZ-treated group (Figure 4).

**Figure 5:**
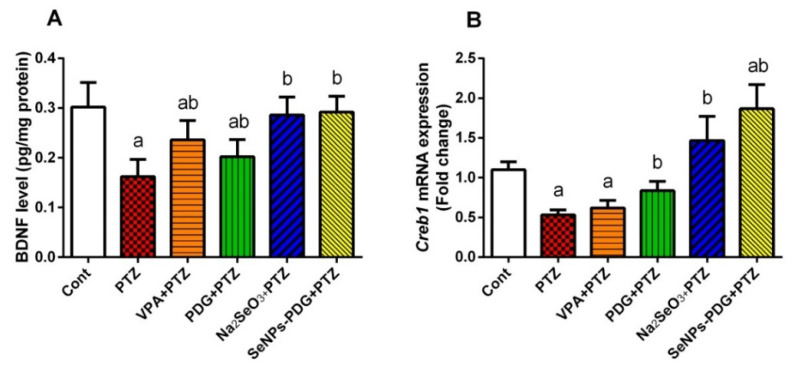
Effects of orally administered prodigiosin (PDG), sodium selenite (Na_2_SeO_3_), and green biosynthesized SeNPs with PDG (SeNPs-PDG) on (**A**) BDNF and (**B**) *Creb-1* mRNA expression levels in hippocampal tissue following pentylenetetrazole (PTZ)-induced epileptic seizures. ^a^ and ^b^ denote significant differences (*p* < 0.05) relative to the untreated control and PTZ-injected groups, respectively. All results are presented as the mean ± standard deviation (SD).

Additionally, as shown in Figure 5, significant reductions were observed in the mRNA expression of *Creb-1* and the level of BDNF in hippocampal tissue from the PTZ-injected group. Pre-treatment with SeNPs-PDG elevated both BDNF and *Creb-1* expression levels compared with PTZ-injected rats, reflecting the ability of SeNPs-PDG to promote neuron growth and neuron maintenance. 

### 3.5. SeNPs-PDG Administration Alters Neurochemical Levels in Hippocampal Tissue Following PTZ Injection

Significant reductions in the hippocampal levels of 5-HT, the 5-HT metabolite 5-HIAA, DA, NE, and GABA, in addition to reduced AChE activity, were reported in PTZ-treated rats, accompanied by increased levels of DA metabolites, including dihydroxyphenylacetic acid (DOPAC), homovanillic acid (HVA), and glutamate, compared with those in the control rats. Pre-treatment with PDG alone or as SeNPs-PDG significantly modulated the levels of examined neurotransmitters in hippocampal tissue during epileptogenesis, as shown in Table 3.

### 3.6. SeNPs-PDG Administration Protects against Histopathological and Immunohistochemical Changes Associated with the Development of Epileptic Seizures

**Figure 6:**
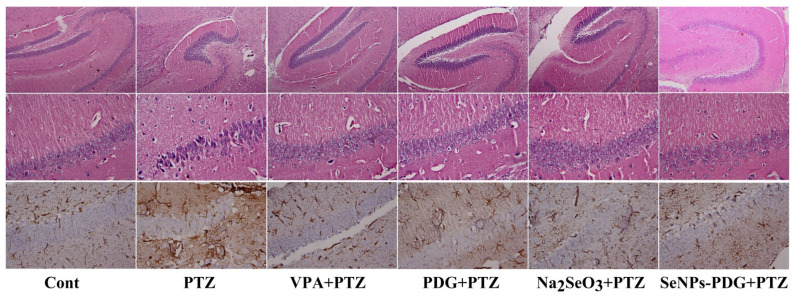
Effect of orally administered prodigiosin (PDG), sodium selenite (Na_2_SeO_3_), and prodigiosin-conjugated with selenium nanoparticles (SeNPs-PDG) on histopathological changes top panel for hippocampus at ×200, middle panel for hippocampus at ×400, and bottom panel for GFAP expression (×400) in the hippocampal tissue following PTZ-induced epileptic seizures.

The control (untreated group) showed normal hippocampal architecture, whereas the PTZ-injected group displayed neuronal degeneration and necrosis associated with pyknotic nuclei in the CA1 region. By contrast, PTZ treatment in rats pretreated with VPA, PDG, Na_2_SeO_3_, and SeNPs-PDG showed near-normal morphology (Figure 6). Additionally, the immunohistochemical examination of the PTZ-treated group showed positive immunostaining for GFAP in the hippocampal tissue, indicating astrocytes activation, supporting the observation of increased inflammatory markers in this group. The experimented groups, especially the SeNPs-PDG-treated group, displayed a marked decrease in GFAP immunoreactivity compared with the PTZ-treated group (Figure 6).

## 4. Discussion

Recently, nanomaterials have received increasing attention as potential and promising co-therapeutic agents against several disorders due to enhanced bioavailability and improved drug delivery to targeted tissues relative to other drug delivery mechanisms [4]. In the present study, we examined the potential neuroprotective and anticonvulsant effects of SeNPs-PDG against PTZ-induced epileptic seizures in rats.

The findings from this study indicated that a single acute dose (60 mg/kg) of PTZ potentiated hippocampal oxidative damage, as characterized by increased ROS production, lipid peroxidation represented by MDA levels, and NO synthesis. By contrast, innate antioxidant molecules, including GSH, GPX, GR, SOD, and CAT, decreased following PTZ injection. At the molecular level, PTZ injection resulted in the downregulation of *Nrf2* mRNA expression. Oxidative stress plays a crucial role in the pathogenesis of epileptic seizures, and neuronal oxidative stress occurs in response to the overproduction of ROS and RNS and the depletion of endogenous antioxidants during status epilepticus [4]. Both ROS and RNS can impair cellular macromolecules and inhibit cellular enzymatic and non-enzymatic antioxidants, eventually resulting in neuronal cell death [35]. Zhu et al. [36] reported that neuronal hyperexcitability associated with epileptic seizures might be attributed to the development of oxidative stress caused by mitochondrial and endoplasmic reticulum dysfunction, resulting in ROS overproduction that exhausts and depletes the cellular antioxidant molecules. Lipid peroxidation occurs when hydroxyl radicals interact with neuronal membrane unsaturated fatty acids, resulting in the formation of lipid peroxide, hydroxide radicals, and MDA [4]. Increased NO levels observed following PTZ-mediated epileptic seizures may be due to the overexpression of iNOS, which is the rate-limiting enzyme involved in NO formation. When produced in large quantities, NO interacts with O_2_^•−^ to produce ONOO^−^, which causes deleterious neurological effects, further supporting our findings [37]. *Nrf2* is a transcriptional mediator that protects against neuronal oxidative insults by regulating the expression and activity of cellular antioxidants. The downregulation of *Nrf2* in the current study may be associated with the observed decrease in cellular antioxidant capacity following PTZ injection [38]. 

However, SeNPs-PDG supplementation prevented PTZ-induced changes in the redox status of hippocampal tissue, as demonstrated by the inhibition of ROS production and MDA and NO formation and the enhancement of *Nrf2* and antioxidant-related protein expression. These findings support the promising neuroprotective and antioxidative properties of SeNPs-PDG. In their earlier study, Chang et al. [39] reported that PDG prevented neuronal oxidative and nitrosative insults induced by hypoxia and ischemia by inhibiting NADPH oxidase2 activity and ROS production. Additionally, PDG suppressed microcystin-LR–mediated oxidative stress in HepG2 cells by inhibiting ROS production and activating *Nrf2* [40]. Moreover, PDG attenuated the development of oxidative damage associated with a gastric ulcer model, as demonstrated by decreased levels of lipid peroxidation and NO production and elevated levels of cellular antioxidant defense system components [20]. This effect may be due to the free radical-scavenging activity of PDG [41].

Selenium is included in the structures of selenoproteins and selenoenzymes, and selenium-based nanoformulations are small in size with large surface areas. These characteristics confer SeNPs with potent antioxidative capacity by allowing for the enhanced scavenging of free radicals, inhibiting the induction of oxidative reactions [42]. Yuan et al. [4] indicated that the administration of SeNPs prior to PTZ injection protected against neuronal oxidative damage by inhibiting MDA, NO, and ROS formation, accompanied by the upregulation of *Nrf2*, in addition to increasing GSH levels and antioxidant enzyme activity in the hippocampus. Additionally, chitosan-stabilized SeNPs displayed neuroprotective activity and attenuated cortical oxidative injury following acrylamide intoxication through the inhibition of lipid peroxidation and NO production, as well as enhancing the pool of available GSH. Moreover, SeNPs combined with metformin alleviated neurobehavioral changes and blunted lipid peroxidation, in addition to activating *Nrf2* and downstream antioxidants (GSH, GPx, GST, SOD, and CAT) in the brain tissue of diabetic model rats [43].

Accumulating evidence has strongly suggested that changes in monoaminergic, amino acidergic, and cholinergic transmission play crucial roles in regulating epileptogenesis [17,20]. In line with previous reports, the current study identified marked decreases in the levels of 5-HT and its metabolite (5-HIAA), DA, and NE and reduced AChE activity in PTZ-injected rats, which was accompanied by apparent increases in the DOPAC and HVA levels. Deficiencies in the levels of biogenic amines have been demonstrated to trigger seizure susceptibility and the development of behavioral deficits and neuropathological deficits in clinical and experimental studies [44,45,46]. Earlier reports showed a disturbance in the synthesis, release, and reuptake of monoamines following the initiation of epileptic seizures [47]. Additionally, excessive ROS generation and neuronal apoptosis triggered neurodegeneration and the depletion of monoamines and their metabolites [48]. Cholinergic dysfunction plays a crucial role in the pathogenesis of epilepsy. AChE hydrolyzes acetylcholine into acetic acid and choline at the synaptic cleft, and a decrease in AChE activity results in the accumulation of acetylcholine, inducing neuronal hyperexcitability and the development of status epilepticus [49]. The enhancement of monoaminergic transmission may also be implicated in the mechanisms of action underlying the efficacy of several clinically prescribed antiepileptic medications [4].

In this study, pre-treatment with SeNPs-PDG demonstrated antiepileptic activity via the modulation of monoaminergic and cholinergic transmission following PTZ injection–mediated status epilepticus. Although the neuroprotective effects of PDG have received little attention, selenium is well known as a neuroprotective agent in either its metal form or as nanoformulations. Similar to antioxidants, experimental treatment with selenium has demonstrated positive responses against various neurological diseases associated with the development of oxidative injury [4,50]. In line with the results of the present study, Yuan et al. [4] reported that SeNPs pre-treatment markedly modulated the levels of monoamines and activated AChE in an epileptic rat model due to antioxidant effects. In addition to antioxidant activity, organoselenium compounds showed antidepressant-like actions by modulating 5-HT, DA, and NE transmission in cortical and hippocampal tissue [51,52]. Additionally, selenium administration attenuated reduction in DA and its metabolites (DOPAC and HVA) in vivo and in vitro following methamphetamine exposure [53,54]. In agreement with the obtained findings, Ji et al. [55] revealed that the neuroprotective impacts of selenium nanoformulations are primarily mediated by the activation of AChE in an Alzheimer’s disease model.

Associated with these changes in neurotransmitters, we also recorded a significant decrease in the GABA contents, accompanied by a marked increase in glutamate levels in hippocampal tissue following PTZ injection. An accepted pathophysiological mechanism associated with epileptogenesis is the imbalance between excitatory and inhibitory neurotransmitters [4]. Glutamate and GABA represent the predominant excitatory and inhibitory neurotransmitters in the central nervous system, respectively. Studies have demonstrated that the induction of tonic and clonic seizures is correlated with the blockade of GABAergic transmission, resulting in neuronal hyperexcitability and action potential discharges [56]. Decreased inhibitory activity has been attributed to the downregulation of GABAA receptors and glutamic acid decarboxylase, the rate-limiting enzyme during GABA synthesis [57]. When released in excessive amounts, glutamate induces neurotoxic effects, including the development of epileptic seizures and neuronal death, through the overactivation of glutamate receptors [58,59]. Therefore, agents that inhibit glutamate release and enhance GABAergic transmission are expected to show antiepileptic activities. In the current study, SeNPs-PDG administration restored the glutamate/GABA balance in the hippocampus, which is reflected by the observed anti-seizure activity in response to PTZ-mediated neuronal hyperexcitability. Sodium selenite was found to protect neurons against excitotoxic insults, ROS production, and apoptotic events in response to glutamate-mediated neurotoxicity [60]. Interestingly, SeNPs administration also increased the GABA levels in the brain following exposure to cypermethrin, which the authors attributed to its antioxidant and anti-inflammatory properties [61].

CREB is a leucine zipper transcription factor that regulates neuronal proliferation, differentiation, and survival and maintains synaptogenesis in the adult brain. Phosphorylated CREB regulates neurotrophic factors, including BDNF [62]. In accordance with previous reports [9], PTZ injection-induced epileptic seizures, accompanied by reduced *CREB* mRNA expression and BDNF levels in hippocampal tissue. The downregulation of CREB and BDNF are strongly associated with the initiation of seizures, and agents that increase these neuronal regulators could be used as antiepileptic drugs [62]. SeNPs-PDG administration increased CREB and BDNF levels compared with PTZ-injected rats, indicating neuroprotective effects. Abd Al Haleem and El-Bakly [63] reported that selenium treatment significantly increased the hippocampal expression of CREB and BDNF in a high fat/high cholesterol diet-mediated model of behavioral and neurological deficits.

The current study revealed that PTZ induced a neuroinflammatory state characterized by high GFAP immunoreactivity, excessive pro-inflammatory cytokine secretion (TNF-α and IL-1β), and the activation of *Nos2*, Cox-2, and NF-κB in hippocampal tissue. Oxidative stress and neuroinflammation reinforce each other during the development of epileptic seizures [64]. ROS overproduction following seizure onset has been suggested to activate NF-κB, further enhancing the release of TNF-α and IL-1β [65]. Moreover, IL-1β upregulates the neuronal mRNA expression of Cox-2 [66], resulting in prostaglandin production, which may play a role in seizure-mediated neuronal excitability and death by stimulating astrocytes to produce glutamate [67]. Furthermore, increased detection of GFAP reflects the proliferation and activation of astrocytes, which cause neurodegeneration and apoptosis by stimulating the release of pro-inflammatory mediators and ROS [68]. We found that SeNPs-PDG downregulated GFAP and inhibited the production of TNF-α and IL-1β while simultaneously preventing *Nos2* and Cox-2 mRNA expression and NF-κB nuclear translocation. The obtained data indicated that SeNPs-PDG attenuates PTZ-induced seizures by modulating neuroglia-mediated inflammatory reactions. PDG and its derivatives showed immunomodulatory effects and decreased elevated pro-inflammatory mediators in an atherosclerosis mouse model. Additionally, PDG derived from a marine sponge-associated actinomycete decreased the levels and expression of various inflammatory markers, including myeloperoxidase, IL-1β, TNF-α, iNOS, Cox-2, and NF-κB in a murine gastritis model [20].

Previous studies have demonstrated that seizures induce transient cerebral ischemia, leading to neuronal death in the brain [69]. We found that PTZ-induced epilepsy in mice was associated with increased cytosolic cytochrome c contents and pro-apoptotic protein expression (Bax and caspase-3), in addition to a decrease in anti-apoptotic protein expression (Bcl-2). Seizures cause both oxidative stress and inflammation, which induce widespread neuronal loss due to ROS and cytokine upregulation. Furthermore, ROS production disrupts mitochondrial Ca^2+^ homeostasis, resulting in the opening of the mitochondrial permeability transition pore and subsequent mitochondrial swelling. Mitochondrial membrane rupture releases cytochrome c into the cytosol, activating caspase-9 and caspase-3, resulting in the activation of various cell death pathways [5]. Thus, administering an antiepileptogenic agent with antioxidant and anti-inflammatory properties is expected to exert beneficial effects by preventing neuronal death in the brain. 

Interestingly, pre-treatment with SeNPs-PDG protected hippocampal neurons, suppressing apoptotic events associated with seizure propagation. The anti-apoptotic activity of PDG was explored previously by Abdelfattah et al. [20], who reported that PDG blocked the apoptotic cascade associated with gastric lesions induced by acidified ethanol injections through the upregulation of Bcl-2 and the downregulation of Bax and caspase-3. In agreement with the results of the present study, Yuan et al. [4] showed that SeNPs administration exhibited anticonvulsant activity through the inhibition of pro-apoptotic protein expression (Bax and caspase-3) and the enhancement of anti-apoptotic protein expression (Bcl-2) in hippocampal tissue. Additionally, selenium and its nanoform suppressed neuronal loss caused by cadmium by decreasing Bax and increasing Bcl-2 [70].

## 5. Conclusions

The findings of this study revealed that SeNPs-PDG presents potent neuroprotective and anticonvulsant activities against PTZ-mediated epileptic seizures in rats through normalizing the behavioral changes associated with the development of epileptic seizures, inhibiting pro-oxidative insults (ROS, NO, and MDA), enhancing antioxidative defense systems (GSH, GPx, GR, SOD, CAT, and *Nrf2*), suppressing neuronal inflammation (TNF-α, IL-1β, Cox2, *Nos2*, and NF-κB), preventing the neuronal apoptosis by decreasing the pro-apoptotic factors and increasing the anti-apoptotic protein, and modulating monoaminergic, aminoacidergic, and cholinergic transmission significantly in the hippocampal tissue. 

## Figures and Tables

**Table 1:** Primer sequences of genes analyzed in Real Time-PCR.

Name	Accession Number	Sense (5′---3′)	Antisense (5′---3′)
*Gapdh*	NM_017008.4	AGTGCCAGCCTCGTCTCATA	TCCCGTTGATGACCAGCTTC
*Nrf2*	NM_031789.2	CAGCATGATGGACTTGGAATTG	GCAAGCGACTCATGGTCATC
*Nos2*	NM_012611.3	GGTGAGGGGACTGGACTTTTAG	TTGTTGGGCTGGGAATAGCA
*Creb-1*	NM_001320793.2	CGAGAACCAGCAGAGTGGAG	TTCACTGACATCCTGCTTTACAAT

The abbreviations of the genes; *Gapdh*: Glyceraldehyde 3-phosphate dehydrogenase; *Nrf2*: Nuclear factor-erythroid 2-related factor 2; *Creb-1*: cAMP responsive element binding protein 1; *Nos2*: Nitric oxide synthase 2.

**Table 2:** Effects of orally administered prodigiosin (PDG), sodium selenite (Na_2_SeO_3_), and green biosynthesized SeNPs with PDG (SeNPs-PDG) on pentylenetetrazole (PTZ)-induced behavioral changes and seizure latency and duration.

Groups	No. of Convulsion/No. of Animals Used	Duration of the Seizure (min)	Flexion (min)	Extension (min)	Clonus (min)
Cont	0/6	0.0 ± 0.0	0.0 ± 0.0	0.0 ± 0.0	0.0 ± 0.0
PTZ	6/6	29.3 ± 2.73 ^a^	7.0 ± 1.41 ^a^	13.17 ± 1.94 ^a^	8.8 ± 0.98 ^a^
VPA + PTZ	1/6	3.3 ± 8.16 ^b^	0.7 ± 1.63 ^b^	1.67 ± 4.08 ^b^	1.2 ± 2.86 ^ab^
PDG + PTZ	4/6	15.3 ± 12.13 ^ab^	4.5 ± 3.56 ^ab^	7.67 ± 6.02 ^ab^	3.4 ± 3.43 ^ab^
Na_2_SeO_3_ + PTZ	3/6	11.0 ± 12.12 ^ab^	2.5 ± 2.81 ^ab^	5.17 ± 5.74 ^ab^	3.3 ± 3.78 ^ab^
SeNPs-PDG + PTZ	1/6	3.7 ± 8.98	1.0 ± 2.45 ^ab^	1.5 ± 3.67 ^b^	1.3 ± 3.27 ^ab^

^a^ and ^b^ denote significant differences (*p* < 0.05) compared with the untreated control and PTZ-injected groups, respectively. All records are presented as the mean ± standard deviation (SD).

**Table 3:** Effects of orally administered prodigiosin (PDG), sodium selenite (Na_2_SeO_3_), and green biosynthesized SeNPs with PDG (SeNPs-PDG) on the levels of monoamines, metabolites, and acetylcholinesterase activity in pentylenetetrazole (PTZ)-treated rats.

Parameters	Cont	PTZ	VPA + PTZ	PDG + PTZ	Na_2_SeO_3_ + PTZ	SeNPs-PDG + PTZ
5-HT (μg/g tissue)	8.76 ± 0.92	4.04 ± 0.64 ^a^	7.21 ± 0.71 ^b^	6.46 ± 0.86 ^ab^	7.55 ± 0.95 ^b^	8.50 ± 1.02 ^b^
DA (μg/g tissue)	0.161 ± 0.02	0.091 ± 0.01 ^a^	0.139 ± 0.02 ^b^	0.121 ± 0.02 ^ab^	0.133 ± 0.02 ^ab^	0.159 ± 0.01 ^b^
NE (μg/g tissue)	0.35 ± 0.04	0.12 ± 0.02 ^a^	0.34 ± 0.05 ^b^	0.18 ± 0.05 ^ab^	0.25 ± 0.04 ^ab^	0.28 ± 0.04 ^b^
GABA	120.3 ± 11.2	45.0 ± 7.8 ^a^	106.8 ± 12.6 ^b^	76.1 ± 12.9 ^ab^	90.8 ± 7.7 ^ab^	117.9 ± 10.1 ^b^
5-HIAA	37.6 ± 4.06	14.3 ± 3.86 ^a^	31.3 ± 5.70 ^b^	20.0 ± 4.38 ^ab^	23.64 ± 4.26 ^ab^	26.8 ± 4.01 ^ab^
DOPAC	2.9 ± 0.77	6.0 ± 0.92 ^a^	2.6 ± 0.70 ^b^	3.9 ± 0.75 ^ab^	3.8 ± 0.76 ^ab^	4.2 ± 0.58 ^ab^
HVA	13.4 ± 2.63	33.77 ± 4.53 ^a^	14.84 ± 5.47 ^b^	30.97 ± 5.17 ^ab^	24.57 ± 5.37 ^ab^	22.35 ± 3.30 ^ab^
Glutamate	617.0 ± 57.4	1122.9 ± 193.4 ^a^	600.5 ± 111.6 ^b^	817.3 ± 113.9 ^ab^	678.2 ± 72.5 ^b^	639.8 ± 58.0 ^b^
AChE activity (μmol/min/mg protein)	6.82 ± 1.35	4.29 ± 1.37 ^a^	7.68 ± 1.38 ^b^	4.95 ± 1.40 ^a^	6.99 ± 1.64 ^b^	6.65 ± 1.27 ^b^

^a^ and ^b^ denote significant differences (*p* < 0.05) compared with the untreated control and PTZ-injected groups, respectively. All results are presented as the mean ± standard deviation (SD).

## Data Availability

Available upon request.

## Supplementary Materials

The following are available online at https://www.mdpi.com/article/10.3390/biology11030354/s1, Figure S1: Morphological shape of SeNPs-PDG as examined by TEM.
