# Supplementary figures and images for: Selenium Nanoparticles with Prodigiosin Rescue Hippocampal Damage Associated with Epileptic Seizures Induced by Pentylenetetrazole in Rats
Source: Biology (Basel). 2022 Feb 23;11(3):354. doi: 10.3390/biology11030354 (PMC8945383; doi:10.3390/biology11030354)

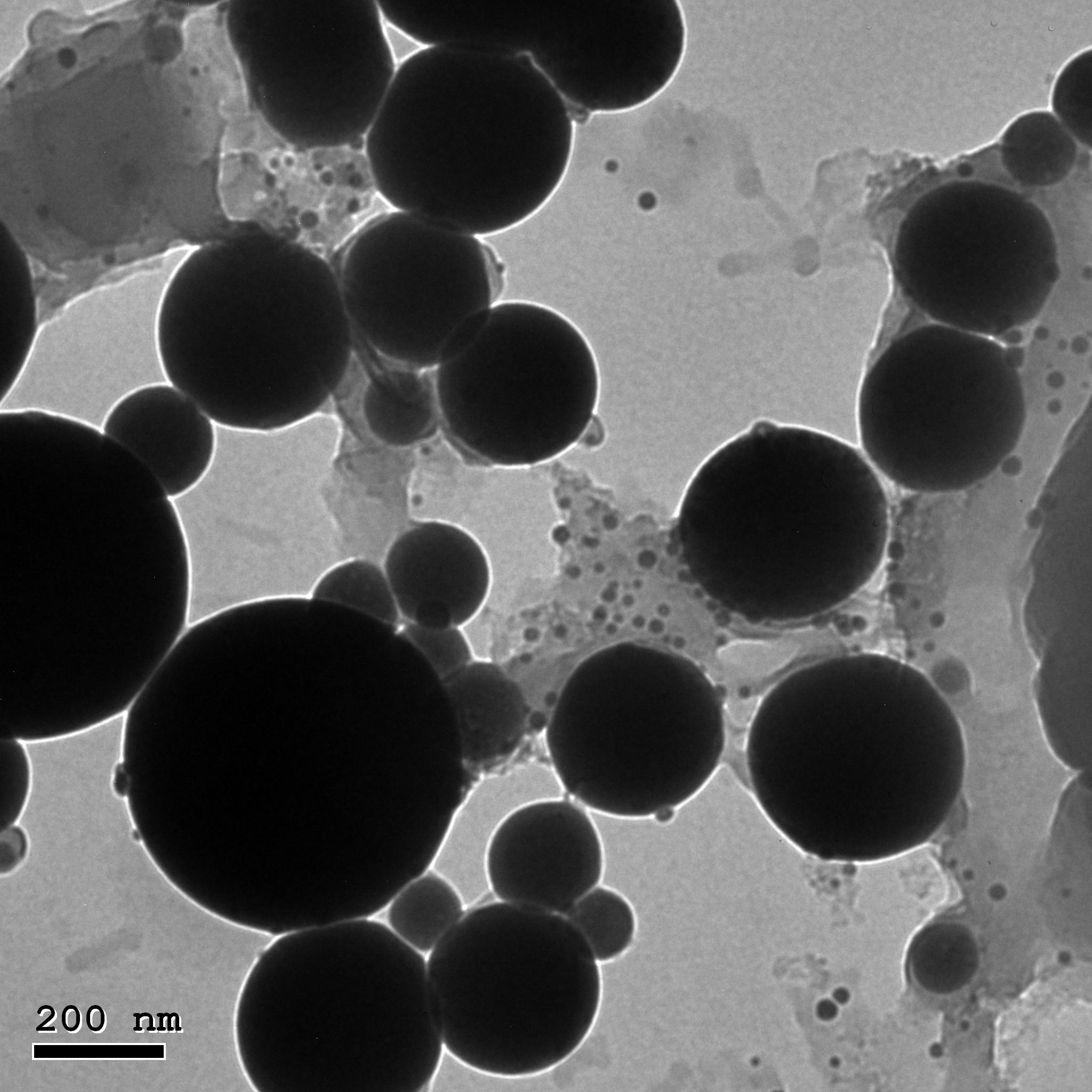

Supplement: Supplementary file 1 [file biology-11-00354-s001.zip › Figure S1.jpg]
